# Zoonotic Relevance of *Toxocara* spp. in North Macedonia: Retrospective Veterinary Findings and a Clinically Confirmed Case of Human Ocular Toxocariasis

**DOI:** 10.3390/pathogens15060595

**Published:** 2026-06-01

**Authors:** Ana Marija Radevska, Bojana Chapkunovska, Katerina Spasovska, Fadil Cana, Stefan Pandilov, Verica Simin, Pavle Banović, Dejan Jakimovski, Aleksandar Cvetkovikj

**Affiliations:** 1 Balkan Association for Vector-Borne Diseases, Branch Skopje—North Macedonia, 1000 Skopje, North Macedonia; anamarija.radevska@medf.ukim.edu.mk (A.M.R.); b.chapkunovska@fvm.ukim.edu.mk (B.C.); kspasovskamk@yahoo.com (K.S.); 2 Department of Parasitology and Parasitic Diseases, Faculty of Veterinary Medicine, Ss. Cyril and Methodius University in Skopje, Lazar Pop-Trajkov 5-7, 1000 Skopje, North Macedonia; 3 University Clinic for Infectious Diseases and Febrile Conditions, 1000 Skopje, North Macedonia; dr.cana@gmail.com; 4 Faculty of Medicine, Ss. Cyril and Methodius University in Skopje, 1000 Skopje, North Macedonia; stefan.pandilov@gmail.com; 5 University Clinic for Eye Diseases, 1000 Skopje, North Macedonia; 6 Pasteur Institute Novi Sad, 21000 Novi Sad, Serbia; vericasimin071@gmail.com (V.S.); pavle.banovic@mf.uns.ac.rs (P.B.); 7 Diagnostics and Laboratory Research Task Force, Balkan Association for Vector-Borne Diseases, 21000 Novi Sad, Serbia; 8 Department of Microbiology with Parasitology and Immunology, Faculty of Medicine, University of Novi Sad, 21000 Novi Sad, Serbia

**Keywords:** *Toxocara* spp., ocular toxocariasis, dogs, cats, North Macedonia

## Abstract

Background: *Toxocara canis* and *Toxocara cati* are zoonotic nematodes of dogs and cats that maintain a human infection risk through environmental contamination with highly resistant eggs. Data on toxocariasis in North Macedonia are limited, and ocular toxocariasis (OT) remains a clinically important but easily overlooked manifestation of human infection. Methods: This retrospective assessment combined coprological data from dogs and cats with a complementary clinical description of a human case of OT. Routine fecal samples from dogs and cats from January 2018 to March 2026 were morphologically examined. The human case of OT was a 13-year-old boy with unilateral ocular disease. Results: Of 465 samples, *Toxocara* spp. eggs were detected in 14, corresponding to an overall detection of 3.0%. Detection was 3.0% in dogs (11/371) and 3.2% in cats (3/94), with no significant difference between species. The human clinical component involved unilateral visual loss, strabismus, and posterior segment inflammatory changes. Conclusions: Dogs and cats in North Macedonia showed sporadic *Toxocara* spp. egg shedding, supporting ongoing zoonotic exposure potential. The clinically confirmed OT case complements the animal data and underscores the need for improved awareness among clinicians and veterinarians, as well as strengthened preventive measures aimed at reducing environmental contamination and zoonotic exposure.

## 1. Introduction

*Toxocara canis* and *Toxocara cati* are common intestinal nematodes of dogs and cats and among the best-recognized zoonotic helminths associated with companion animals [1,2,3]. Their public health relevance derives less from severe disease in the definitive host than from the large number of eggs shed into the environment and the long-term persistence of infective eggs, for over 2 years, in soil and contaminated outdoor spaces [4,5,6]. For humans, especially children and people with frequent contact with animals or soil, exposure usually occurs through accidental ingestion of embryonated eggs from contaminated hands, food, water, or fomites [1,4,5].

The global scale of this zoonotic threat is reflected in pooled prevalence estimates of approximately 11.1% for *T. canis* in dogs and 17% for *T. cati* in cats, corresponding to more than 100 million infected dogs worldwide [7,8]. Similar findings have also been reported across the Balkan region, documenting ongoing *Toxocara* spp. circulation in animals and environmental contamination of public areas [9,10,11,12,13]. In North Macedonia, available veterinary data on *Toxocara* spp. circulation remain limited, although previous studies have documented the presence of the parasite in stray and shelter dogs [14,15].

Human toxocariasis (HT) encompasses a spectrum of syndromes related to larval migration, including visceral, neurologic, covert, and ocular disease [1,3]. Ocular toxocariasis (OT) is an important but probably under-recognized manifestation that typically presents as unilateral visual loss, strabismus, posterior or peripheral retinal granuloma, or endophthalmitis-like inflammation [16,17,18]. The diagnosis of OT is generally clinical with serological support rather than direct parasitological confirmation [16,17,18]. Delayed recognition may result in irreversible ocular damage even when systemic laboratory findings, including eosinophilia, are absent [18,19,20]. OT occurs predominantly in children, with reported mean ages at presentation ranging from approximately 6.7 to 14.3 years [3,21].

Human exposure to *Toxocara* spp. has been documented across Europe and represents an under-recognized zoonotic disease burden [22,23]. Within the Balkan region, serological evidence of human exposure has been reported in several countries, including Bulgaria, Greece, Romania, Serbia, and Slovenia [24,25,26,27,28], while clinically confirmed cases of OT have previously been documented in Serbia and Slovenia [28,29]. To the best of our knowledge, there have been no previously documented cases of HT in North Macedonia. In this context, animal surveillance and clinically characterized human cases represent complementary observations of the zoonotic relevance of *Toxocara* spp.

This study aimed to determine the prevalence of *Toxocara* spp. in veterinary diagnostic submissions from dogs and cats in North Macedonia using retrospective coprological data and to describe a clinically confirmed case of human OT as a complementary human-health observation.

## 2. Materials and Methods

### 2.1. Study Design

This retrospective study included two complementary components: (i) archived coprological diagnostic data from dogs and cats and (ii) clinical, ophthalmological, and serological data from one patient with OT. The animal component covered the period from January 2018 through March 2026 and was based on samples submitted for routine diagnostic evaluation. The human clinical component was based on the patient’s infectious diseases and ophthalmological records, with follow-up after antiparasitic treatment.

### 2.2. Animal Samples and Inclusion Criteria

The animal dataset comprised fecal samples from dogs and cats submitted to the Laboratory for Parasitology and Parasitic Diseases, Faculty of Veterinary Medicine, Skopje, North Macedonia. Samples originated from veterinary practices and owners in North Macedonia and were submitted as part of routine diagnostic work-ups intended to confirm or exclude intestinal parasitic infection. The indication for submission was not systematically available in the archived records; therefore, the cohort should be interpreted as routine diagnostic submissions rather than a defined symptomatic or asymptomatic population.

Fecal samples from dogs or cats originating from North Macedonia were included in the study. To ensure data independence, each laboratory submission was cross-referenced using unique patient identifiers and owner records at the time of admission. To reduce duplicate counting, only the first available sample per individual animal was included when this could be determined from the laboratory records, whereas repeated post-treatment samples were excluded when identifiable. Samples originating outside North Macedonia were excluded. Metadata on age, sex, lifestyle, and deworming history were not consistently available and therefore could not be used as analytical variables.

Samples were collected by owners or clinicians into sterile containers and transported to the laboratory under refrigerated conditions. On arrival, samples were processed immediately or stored at 4 °C and examined within 24 h.

### 2.3. Parasitological Examination of Animal Fecal Samples

All submitted samples underwent macroscopic and microscopic evaluation. Macroscopic examination included assessment of color, odor, and fecal consistency, as well as screening for visible blood, mucus, pus, or adult parasites. Fecal consistency was scored according to the Purina 1–7 scale, where lower values represent firmer stool and higher values represent soft to liquid feces.

Microscopic examination was performed via direct fecal smear and a centrifugal flotation procedure using a laboratory-prepared zinc sulfate solution (specific gravity 1.2), following established veterinary parasitology protocols. Preparations were examined using a light microscope (Olympus BX43, Olympus Corporation, Tokyo, Japan) at 40×, 100×, and 400× magnification. *Toxocara* spp. eggs were identified morphologically to the genus level according to standard veterinary parasitology criteria; no species-specific or molecular confirmation was performed [30].

### 2.4. Analysis Implemented for OT in Human

The human clinical component involved infectious diseases and ophthalmological evaluation of a child referred with suspected OT. Clinical assessment included history taking, physical examination, and review of prior medical documentation. Ophthalmological assessment included visual acuity testing, slit-lamp examination, fundus examination, and available ocular imaging from the referral work-up. Differential diagnostic considerations included OT and ocular toxoplasmosis.

Laboratory testing included ELISA detection of anti-*T. canis* IgG using the Toxocara-IgG-EIA-BEST kit (Cat. No. 2752, Vector-Best, Novosibirsk, Russia). Because serology alone is insufficient for case definition, a serum sample was analyzed via confirmatory Western blot testing (LDBIO Diagnostics, Lyon, France; Cat No. TXA-WB12G). The working case definition for OT required compatible unilateral ocular findings together with positive anti-*T. canis* serology and confirmatory Western blot results, with alternative diagnoses considered on the basis of the available clinical data.

### 2.5. Statistical Analysis

Statistical analysis was performed using GraphPad Prism version 10 (GraphPad Software, San Diego, CA, USA). Prevalence of *Toxocara* spp. in animal samples was calculated as the percentage of positive samples among all examined samples, overall and stratified by species. Exact binomial 95% confidence intervals (Clopper–Pearson method) were calculated for all prevalence estimates. Differences in prevalence between dogs and cats and between geographic groups were assessed using Fisher’s exact test. Relative risk with 95% confidence intervals was calculated from 2 × 2 contingency tables. A two-sided *p*-value < 0.05 was considered statistically significant. Annual and monthly distributions were summarized descriptively and were not subjected to inferential species comparison. Because the submissions formed an unstandardized convenience sample, with small and unequal denominators and a geographic composition that varied between years, year- or season-stratified comparisons between dogs and cats would not be epidemiologically interpretable. The pooled species and geographic comparisons over the whole study period were treated as exploratory.

## 3. Results

### 3.1. Animal Sample Dataset

Between January 2018 and March 2026, 466 fecal samples from dogs and cats were submitted to the Laboratory for Parasitology and Parasitic Diseases for routine diagnostic evaluation. One sample originating outside North Macedonia was excluded. The final analytical dataset therefore comprised 465 samples, including 371 and 94 from dogs and cats, respectively.

Available fecal consistency records indicated that most samples were soft to liquid. Purina scores 5–7 were reported for 429 samples, whereas lower scores were less frequent (score 4, *n* = 11; score 3, *n* = 17; score 2, *n* = 7). No macroscopic blood, mucus, pus, or visible adult parasites were recorded in the submitted specimens.

### 3.2. Overall Prevalence of Toxocara spp. in Dogs and Cats

*Toxocara* spp. eggs were detected in 14 of 465 samples, corresponding to an overall prevalence of 3.0% (95% CI 1.7–5.0). Among dogs, 11 of 371 samples were positive (3.0%; 95% CI 1.5–5.2), compared with 3 of 94 cat samples (3.2%; 95% CI 0.7–9.0). There was no statistically significant difference in positivity between dogs and cats (RR = 0.93; 95% CI 0.26–3.26; Fisher’s exact test *p* > 0.9999). Because the diagnosis was based on egg morphology, all positive animal findings were reported at the genus level as *Toxocara* spp.

### 3.3. Annual Distribution by Species

Annual prevalence estimates showed no consistent temporal trend throughout the study period (Table 1). In dogs, annual prevalence generally ranged from 2.0% to 4.3%, with no positives recorded in the partial 2026 dataset. In cats, higher annual percentages were observed in 2021, 2023, and 2025, but these values were based on very small sample numbers and should not be overinterpreted. Annual figures are presented descriptively only; the year-stratified denominators were too small and the underlying submissions too heterogeneous to support a valid species comparison within individual years.

### 3.4. Monthly Distribution

Positive samples were detected in several months without an obvious seasonal pattern. Aggregate monthly positivity was recorded in January (2/55; 3.6%), February (1/48; 2.1%), March (1/37; 2.7%), April (1/35; 2.9%), August (2/29; 6.9%), October (2/53; 3.8%), November (3/46; 6.5%), and December (2/38; 5.3%). No positive samples were identified in May, June, July, or September.

When examined by host species, dog-positive samples were distributed across January, February, March, April, August, November, and December, whereas cat-positive samples were limited to October (2/10; 20.0%) and November (1/13; 7.7%). Because the number of positive samples was small and monthly counts were sparse, no formal statistical comparison across months was made.

### 3.5. Geographic Distribution

**Figure 1:**
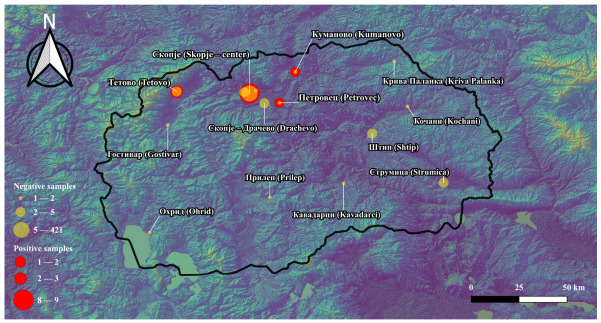
Geographic distribution of submitted samples and *Toxocara* spp.-positive findings by region. Red bubble size is proportional to the number of positive samples, and a yellow bubble marks the location where negative samples were acquired. The 293 background terrain visualization was obtained with the Copernicus Global Digital Elevation Model (GLO–30), accessed through OpenTopography and processed in QGIS using hillshade and pseudocolor elevation rendering.

Sample submissions were highly concentrated in the Skopje region, accounting for 444 of 465 samples (95.5%). The remaining 21 samples originated from seven other statistical regions: Polog (*n* = 7), Southeastern (*n* = 4), Eastern (*n* = 4), Northeastern (*n* = 3), Vardar (*n* = 1), Southwestern (*n* = 1), and Pelagonia (*n* = 1) (Figure 1).

Most positive samples originated from the Skopje region (10/14; 71.4%), followed by Polog (3/14; 21.4%) and the Northeastern region (1/14; 7.1%) (Figure 1). All positive cat samples were from Skopje. This difference reached statistical significance (Fisher’s exact test, *p* = 0.0023); however, these findings should be interpreted cautiously because of the small sample size of the non-Skopje group.

### 3.6. Clinical Component: Human OT Case

A 13-year-old boy from a rural area in the Polog region (Tetovo) was referred in December 2024 for evaluation of suspected OT after approximately four months of progressive darkening of the visual field and decreased vision in the left eye. According to the family, left-eye asymmetry and bluish discoloration had first been noticed during a stay in Albania, where OT and toxoplasmosis were considered in the differential diagnosis, and empirical treatment with trimethoprim–sulfamethoxazole and corticosteroids had already been started.

**Figure 2:**
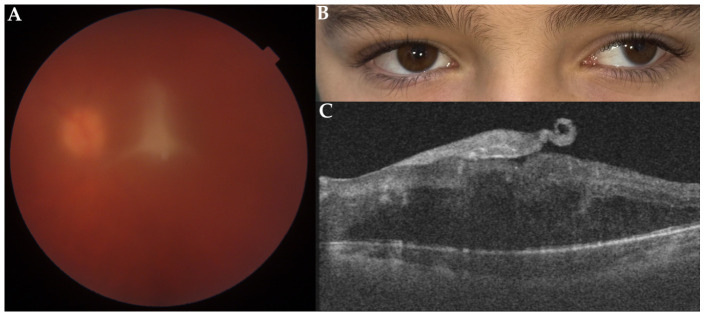
Clinical component of the human OT case: (**A**) Fundus photograph of the left eye, vitreous haze 2+, central posterior granuloma in the maculo-papillary region with fibrous membrane over it. (**B**) Exotropia of the left eye as a consequence of persistent poor vision in the eye due to pathological changes in the macular region. (**C**) Optical coherence tomography of the macula, epiretinal dense fibroglial membrane destruction of inner retinal layers, and intraretinal fluid diffused into outer retinal layers.

On reassessment in Skopje, the patient had left strabismus (Figure 2B). Best corrected visual acuity in the right eye was 1.0, whereas the left eye had severely reduced vision, initially limited to counting fingers. Slit-lamp examination showed a posterior subcapsular cataract and a moderate cellular reaction in the anterior vitreous. Fundus examination revealed a hyperemic optic disc, edema of the maculo-papillary region, and a preretinal gliotic membrane, while the peripheral retina was described as unremarkable (Figure 2A). Optical coherence tomography of the posterior segment was also performed, where a fibrogliotic epiretinal membrane, impaired neurosensory retinal architecture, and intraretinal diffuse fluid were observed (Figure 2C).

The patient was afebrile and in good general condition. Complete blood count, serum biochemistry, and inflammatory markers were within normal limits, and peripheral eosinophilia was absent. ELISA testing demonstrated anti-*T. canis* IgG antibodies, and confirmatory Western blot testing was also positive. A diagnosis of unilateral toxocariasis of the left eye in the form of a central posterior granuloma was made. Exposure history revealed previous close contact with a household dog more than three years before symptom onset, frequent contact with community dogs while playing outdoors, and current residence in a household that kept a cat. Review of earlier documentation showed that a cataract of the left eye had already been recorded approximately 18 months before the present evaluation during a school ophthalmologic screening, but no follow-up had occurred at that time.

After confirmation of the diagnosis, the patient received albendazole 400 mg twice daily for four weeks, together with a short tapering course of oral prednisolone. At follow-up 10 days after treatment initiation, the patient reported slight subjective improvement, and left visual acuity improved to 0.05; however, subsequent ophthalmological follow-up showed no further meaningful improvement. Because of persistent visual impairment due to a complicated cataract, phacoemulsification with intraocular lens implantation was performed eight months after completion of antiparasitic treatment.

## 4. Discussion

### 4.1. Veterinary Findings and Epidemiological Implications

This study provides retrospective veterinary coprological findings on *Toxocara* spp. detection in dogs and cats in North Macedonia, together with a clinically confirmed case of human OT. *Toxocara* spp. eggs were sporadically detected in routine fecal submissions from dogs and cats over the study period, whereas the human case represents the first clinically documented OT case in North Macedonia.

The animal component should be interpreted primarily as evidence of circulation rather than as a population-based prevalence survey, given the retrospective design, diagnostic submission-based sampling approach, and strong geographic concentration of submissions. Even so, an overall positivity of approximately 3% in routine laboratory submissions from owned dogs and cats is epidemiologically relevant, although this figure should not be interpreted as a prevalence estimate for the general companion animal population in North Macedonia. This relatively low prevalence may reflect the characteristics of animals represented in routine veterinary submissions and may differ from rates observed in stray or shelter populations [14,31,32]. By comparison, local cross-sectional data show that stray and shelter dogs in North Macedonia suffer substantially higher infection rates due to continuous environmental exposure and a lack of systematic anthelmintic control [14]. In Serbia, for instance, kenneled and stray dogs carry a heavy parasitic burden, reaching *T. canis* detection rates of 13.7% in public shelters; yet, household pets still exhibit an 11.5% prevalence that escalates to nearly 38.0% among dogs frequenting public parks [9,33]. In Bulgaria, this stratification is equally pronounced: shelter dogs experience intense parasite transmission, resulting in an overall gastrointestinal parasite prevalence of 64.5% [12], whereas client-owned companion animals maintain a stable *T. canis* baseline of 17.76% [34]. Similarly, canine cohorts in suburban and rural regions of Tirana, Albania, exhibit a striking 75.7% *T. canis* prevalence, contrasting sharply with a controlled baseline of roughly 8.0% in companion animals [35]. Finally, multi-population audits in Greece reveal a comparable companion animal baseline of 7.6% in dogs and 8.3% in cats [11]. This broader regional reality demonstrates that low prevalence does not equate to negligible risk. A limited number of egg-shedding animals may contribute to environmental contamination, particularly because adult *Toxocara* worms have high reproductive output and the eggs persist for prolonged periods once they become infective [4,5,6]. Consistent with this concern, environmental studies from different geographic settings have documented *Toxocara* spp. egg contamination in public parks and recreational areas, including detection rates of 8.9% in public spaces in Argentina and widespread contamination across parks in the United Kingdom and Ireland, where *Toxocara* spp. eggs were detected in 86.6% of surveyed parks [36,37]. Additionally, modeling studies suggest that both owned and stray animals can substantially contribute to persistent environmental egg contamination [4].

Temporal and geographic patterns should be interpreted cautiously. Positive samples were distributed across multiple years and several months, but there was no convincing annual or seasonal pattern. Apparent peaks among cats in selected years were driven by very small denominators rather than a stable epidemiologic signal. Likewise, the geographic dataset was heavily skewed toward the Skopje region, which accounted for the overwhelming majority of submissions. Although non-Skopje regions showed a numerically higher proportion of positives, this finding is based on sparse sampling outside the capital and should not be generalized to regional risk without more balanced surveillance.

### 4.2. Clinical and Public Health Implications of OT

The addition of the clinical component in this study illustrates the public-health relevance of *Toxocara* exposure. The patient’s symptoms and ophthalmological findings represent a recognizable pattern compatible with OT manifesting as posterior granulomatous or inflammatory ocular disease [17,18,38,39]. The patient’s history of rural residence and frequent contact with dogs and cats was also compatible with known exposure pathways, including contact with potentially contaminated soil in environments where regular animal deworming may be inconsistent. While other manifestations of HT can be associated with detectable eosinophilia, the absence of eosinophilia in OT is very common, as is observed in our case [18,19,20].

The diagnosis of OT depends strongly on clinician awareness of its characteristic manifestations and a detailed exposure history. Accordingly, OT remains primarily a clinical diagnosis based on compatible findings and exclusion of differential diagnoses, whereas serological findings serve as a supportive tool [16,18]. Although ocular toxoplasmosis and OT have distinct characteristic features, both may present with overlapping posterior segment inflammatory manifestations, highlighting the importance of early consideration of both infections during differential diagnosis [40]. Subsequently, the combination of unilateral specific ocular findings, positive anti-*T. canis* ELISA, and confirmatory Western blot provided strong supportive evidence for the diagnosis of OT. Western blot is particularly valuable when used as a confirmatory test after ELISA because of its higher specificity and its role in strengthening interpretation in diagnostically challenging cases [16,18].

There is no universally accepted therapeutic regimen for OT, and reported management strategies vary according to disease stage, extent of inflammation, and ocular complications [18,41,42,43,44]. Albendazole combined with corticosteroids is a frequently used approach intended to address both the parasitic and inflammatory components of disease resulting from the albendazole-induced parasite degradation [18]. Visual outcome is often variable, especially in chronic or structurally advanced cases. The limited functional recovery in this patient was likely related to delayed diagnosis and established ocular complications, including cataract and fibrogliotic changes, rather than to the absence of anti-inflammatory or antiparasitic treatment alone. The subsequent need for cataract surgery is consistent with the recognized importance of managing late sequelae of ocular inflammation [18,45,46].

From a One Health perspective, the study indicates that the veterinary, medical, and public health observations must be interpreted together. Prevention of HT depends on regular deworming of dogs and cats, hygienic disposal of feces, and hand hygiene after soil or animal contact [4,5]. Clinician awareness is essential because OT presents as an isolated unilateral visual loss without classic systemic laboratory abnormalities, and its fundoscopic features may mimic other posterior uveitides, increasing the risk of misdiagnosis [18]. Collaboration between veterinarians, physicians, ophthalmologists, public health professionals, and pet owners is therefore central to risk reduction.

This study has several limitations. First, the animal component was retrospective and based on routine diagnostic submissions rather than population-based sampling, which limits generalizability. Because samples were submitted for routine diagnostic purposes, the study population may have been biased toward animals with gastrointestinal signs or suspected parasitic infections, potentially influencing the observed prevalence estimates. This selection bias is further compounded by a relatively small sample size spanning eight years, which precludes these findings from serving as a definitive nationwide baseline. Second, submissions were strongly concentrated in the Skopje region, leaving other regions underrepresented. Third, metadata on animal age, sex, clinical status, deworming history, housing, and lifestyle were not consistently available. Fourth, the number of positive samples was low, limiting subgroup analyses. Because the dataset was an unstandardized convenience sample whose composition varied across years and regions, year- and season-stratified comparisons between species were not epidemiologically valid and were restricted to descriptive presentation. Fifth, microscopy may underestimate true prevalence because egg shedding can be intermittent [6]. Sixth, morphology alone does not reliably distinguish *T. canis* from *T. cati* eggs, and no molecular confirmation was performed. While this precludes species-specific tracking or the definitive exclusion of mechanical egg transit via coprophagia, genus-level identification remains the operational standard for routine veterinary coprology. Furthermore, no environmental sampling was performed, limiting assessment of potential soil contamination and environmental transmission pathways relevant to HT. Finally, the human case complements the animal findings conceptually but cannot establish a direct epidemiological link with the sampled pet population or a specific environmental source.

In conclusion, this study provides evidence of *Toxocara* spp. circulation in companion dogs and cats in North Macedonia and documents a clinically relevant case of human OT. Together, these findings support the continuous zoonotic relevance of this pathogen and indicate the need for improvement of awareness among both clinicians and veterinarians, as well as strengthened preventive measures aimed at reducing environmental contamination and zoonotic exposure.

## Figures and Tables

**Table 1:** Annual distribution of *Toxocara* spp. prevalence in dogs and cats.

Year	Species	Samples (*n*)	Positive (*n*)	Prevalence %, 95% CI
2018	Dog	44	1	2.3 (0.06–12.02)
	Cat	9	0	0.0 (N/A)
2019	Dog	51	2	3.9 (0.48–13.46)
	Cat	12	0	0.0 (N/A)
2020	Dog	38	1	2.6 (0.07–13.81)
	Cat	8	0	0.0 (N/A)
2021	Dog	42	1	2.4 (0.06–12.57)
	Cat	10	1	10.0 (0.25–44.50)
2022	Dog	52	2	3.8 (0.46–12.98)
	Cat	15	0	0.0 (N/A)
2023	Dog	49	1	2.0 (0.05–10.85)
	Cat	14	1	7.1 (0.18–33.87)
2024	Dog	47	2	4.3 (0.52–14.53)
	Cat	11	0	0.0 (N/A)
2025	Dog	35	1	2.9 (0.07–14.92)
	Cat	12	1	8.3 (0.21–38.48)
2026 *	Dog	13	0	0.0 (N/A)
	Cat	3	0	0.0 (N/A)
Total	Dog	371	11	3.0 (1.49–5.23)
	Cat	94	3	3.2 (0.66–9.04)

* Data for 2026 include samples collected through March only. N/A = confidence interval not calculated because no positive samples were detected.

## Data Availability

All data generated in this manuscript are available in the main text.

## Abbreviations

The following abbreviations are used in this manuscript:

OT	Ocular toxocariasis
ELISA	Enzyme-linked immunosorbent assay
HT	Human toxocariasis
